# More than hemoglobin – the unexpected diversity of globins in vertebrate red blood cells

**DOI:** 10.14814/phy2.12284

**Published:** 2015-02-03

**Authors:** Miriam Götting, Mikko Nikinmaa

**Affiliations:** 1 Zoological Institute and Zoological Museum, Biocenter Grindel, University of HamburgHamburg, Germany; 2 Department of Biology, Laboratory of Animal Physiology, University of TurkuTurku, Finland

**Keywords:** Gene transcription, globin X, globins, hemoglobin, red blood cells

## Abstract

In many multicellular organisms, oxygen is transported by respiratory proteins, which are globins in vertebrates, between respiratory organs and tissues. In jawed vertebrates, eight globins are known which are expressed in a highly tissue-specific manner. Until now, hemoglobin (Hb) had been agreed to be the only globin expressed in vertebrate erythrocytes. Here, we investigate for the first time the mRNA expression of globin genes in nucleated and anucleated erythrocytes of model vertebrate species by quantitative real-time reverse transcription PCR (qRT-PCR). Surprisingly, we found transcripts of the whole gnathostome globin superfamily in RBCs. The mRNA expression levels varied among species, with *Hb* being by far the dominant globin. Only in stickleback, a globin previously thought to be neuron-specific, *neuroglobin*, had higher mRNA expression. We furthermore show that in birds transcripts of *globin E*, which was earlier reported to be transcribed only in the eye, are also present in RBCs. Even in anucleated RBCs of mammals, we found transcripts of myoglobin, neuroglobin, and cytoglobin. Our findings add new aspects to the current knowledge on the expression of globins in vertebrate tissues. However, whether or not the mRNA expression of these globin genes has any functional significance in RBCs has to be investigated in future studies.

## Introduction

Respiratory proteins, globins in vertebrates, increase the efficiency of oxygen storage and transport since many multicellular organisms cannot obtain enough oxygen to sustain active metabolism by diffusion (Hardison 1996). Globins are well-characterized proteins and used as models for investigating mechanisms of protein evolution, structure, and function (e.g., Dickerson and Geis 1983; Hardison 2012). In vertebrates, they are molecules of 16–23 kDa with a heme prosthetic group, by which they reversibly bind O_2_ and other gaseous ligands. Members of the globin superfamily are widespread among bacteria, fungi, plants, invertebrates, and vertebrates (Hardison 1996). In vertebrates, a total of eight different globins have been described. Phylogenetic analyses revealed that at least two distinct globin lineages emerged prior to the divergence of Protostomia and Deuterostomia. While one lineage comprises the vertebrate-specific globins [hemoglobin *α* (Hb*α*), hemoglobin ß (Hb*β*), myoglobin (Mb), cytoglobin (Cygb), globin E (GbE), globin Y (GbY)], the other one includes neuroglobin (Ngb), globin X (GbX), androglobin (Adgb), and some invertebrate nerve-specific globins (Blank and Burmester 2012; Hoogewijs et al. 2012; Storz et al. 2013). Globins are expressed in a highly species- and tissue-specific manner and have putatively divergent functions (recently reviewed in Burmester and Hankeln 2014). They are all putatively involved in cellular processes related to O_2_ transport and storage, detoxification of reactive oxygen and nitrogen species, sensing and signaling (Vinogradov and Moens 2008) or may have yet unknown functions. Until now, Hb, which is responsible for O_2_ and CO_2_ transport, is the most widely distributed globin and is thought to be the only globin expressed in vertebrate erythrocytes or red blood cells (RBC). Mature erythrocytes of almost all fish, amphibian, reptile, and bird species are oval-shaped and retain their nucleus and all organelles throughout their lifetime (Nikinmaa 1990). In contrast, definite circulating mammalian erythrocytes lack nuclei and organelles. Vertebrate RBCs are tightly packed with Hb and the cellular Hb concentration increases from agnathans to birds and mammals (Snyder and Sheafor 1999). In all jawed vertebrates (gnathostomates) Hb is a tetramer composed of four (2 alpha and 2 beta) globin chains. In mature mammalian RBCs large amounts of *Hb* mRNA, finally comprising over 95% of total cellular mRNA, are accumulated in the cell during erythroid development (Bastos et al. 1977).

None of the recent studies on vertebrate globins (e.g., Kugelstadt et al. 2004; Fuchs et al. 2006; Blank et al. 2011b) have systematically examined circulating erythrocytes for the presence of other members of the globin superfamily (Mb, Ngb, Cygb, GbE, GbY, and GbX). To fill this gap, we collected peripheral blood from representative model vertebrate species with nucleated RBCs (fish, amphibians, birds) and from two mammalian species with anucleated RBCs (mouse, human). By quantitative RT-PCR, we compared globin transcript levels in RBCs with mRNA expression levels in other tissues, such as liver, brain, eyes, and gills in the respective species.

## Materials and Methods

### Blood sample collection

Blood samples of different vertebrate species (three-spined stickleback, *Gasterosteus aculeatus* Linnaeus, 1758; clawed frog, *Xenopus laevis* (Daudin, 1802); chicken, *Gallus gallus* (Linnaeus, 1758)*,* and mouse *Mus musculus*, Linnaeus, 1758) were collected from anesthetized animals and stored in 1 × phosphate-buffered saline (PBS: 140 mmol/L NaCl, 2.7 mmol/L KCl, 8.1 mmol/L Na_2_HPO_4_, 1.5 mmol/L KH_2_PO_4_, pH 7.4) containing 9 mmol/L disodium EDTA. Human blood samples were taken from one of the authors (M.G.) into EDTA and heparin Vacutainers (Sarstedt). Blood was centrifuged at low speed (800 × g) at 4°C for 10 min and plasma was removed. The pelleted RBCs were washed three times in cold PBS containing 9 mmol/L disodium EDTA. In each step, the top layer of RBC was removed to minimize contamination with, for example, white blood cell. Washed RBCs were resuspended in 500 *μ*L 1 × PBS and stored at −80°C until further use. Tissue samples of the respective animals were collected and cut in small pieces, washed in ice cold 1 × PBS to remove blood, and stored in RNAlater at −80°C until further use. All procedures described are in accordance with the guidelines for animal care and experimentation and were approved by the Authority of health and consumer protection (Behörde für Gesundheit und Verbraucherschutz), Hamburg, Germany.

### RNA isolation and cDNA synthesis

Total RNA was isolated from blood and tissue with peqGOLD TRIfast™ (Peqlab, Erlangen, Germany) in combination with the innuPREP RNA Mini Kit including an on-column digestion with RNase-free DNase (Qiagen, Hilden, Germany). RNA was quantified spectrometrically (Nanodrop ND 100, Thermo Scientific, Bonn, Germany) and integrity was checked by formaldehyde agarose gel electrophoresis. Reverse transcription of 0.75 to 1 *μ*g total RNA was performed using the RevertAid First Strand cDNA Synthesis Kit (Thermo Scientific, Bonn, Germany) with oligo-(dT)_18_ primers in a total volume of 20 *μ*L.

### Quantitative real-time reverse transcription PCR

Quantitative real-time reverse transcription PCR (qRT-PCR) was carried out using an ABI 7500 Real-Time PCR system. The cDNA products were amplified in triplicates. Each reaction mixture contained 11 *μ*L SybrGreen Master Mix (Applied Biosystems, Darmstadt, Germany), 1 *μ*L cDNA template (diluted 1:3), 0.5 *μ*L of forward and reverse primers (0.5 mmol/L final concentration), and 9 *μ*L DNase-free water. qRT-PCR primers are available from Table1. A two-step cycling protocol was applied: 10 min at 95°C followed by 40 cycles of 15 sec at 95°C and 1 min at 60°C and 30 sec at 72°C. Specificity of primers and amplification was evaluated using dissociation curves with a temperature range from 60 to 95°C. Standard curve reactions were performed using 10-fold dilutions (10^8^ to 10^2^) of recombinant plasmids (pGEM-T; Promega, Mannheim, Germany) in duplicates. The absolute mRNA copy number was determined by extrapolating the *C*_*t*_ value for each sample on the standard curve and was expressed as copy number per *μ*g RNA (copy no. per *μ*g RNA). Data are analyzed with the 7500 System Sequence Detection Software 2.0.6 and GraphPad Prism6 (GraphPad Software, La Jolla, CA) and expressed as means ± SEM.

**Table 1:** Primers used in quantitative real-time reverse transcription PCR (qRT-PCR) and amplicon size

Species	Transcript	Globin primers (5′–3′)	Amplicon size (nt)
Stickleback	GbX	F: GTCAGGTTGTTTGAGACCCACCC	121
		R: ACATGACCCTGAGGCCGTGCG	
	Ngb	F: CCAGGCTGTTTGAGCTGGACCC	102
		R: GATCCAGGAACTCGGGGCTGGC	
	Cygb2	F: GCAGCCAACTTCGACAACACGC	87
		R: GACGACACCTTCTCCGGGTCATG	
	Hba	F: CGGCCGGACTGTGATGGCTG	188
		R: GAGCCACAGCAGCCAGGAAC	
Clawed frog	GbX	F: GGCTCATGGGCTGAGGGTCC	106
		R: GTAGTGGCTTCTGCCAAGGTCC	
	Ngb	F: GCTGTGAGCAGTCTGGACAGC	143
		R: CCTAAACCCGACTCCAGAGCG	
	GbY	F: GGGAAGTCGTGAGCAGTGCT	159
		R: CCCAGGATTTACAGTGCTCTGGAG	
	Cygb	F: GCACATGGAGGACCCTTTGGAG	89
		R: CCACCACACTGTTGACAGCACCC	
	Hba	F: TCCATAGCCGCTCATGGCGAC	211
		R: GGTCGCTCAGCTTGCTCATGC	
Chicken	Ngb	F: AGGAGTGCCTGGCTGCC	144
		R: CACCAACAGCCTGATGCTTC	
	Cygb	F: GGTCTCCTCCGTTCTGGCCCT	153
		R: GTCCACGCGCCGTGCGCCTC	
	GbE	F: GCAGTGCTGGTCAGGATGTT	104
		R: CTCTGACCTGATCCGACTG	
	Mb	F: CCATCTGGGGAAAAGTGGAGGC	177
		R: GGGTGAGGACAGTAGCTCCATG	
	Hba	F: TCGCCGGCCATGCTGAGGAGT	145
		R: GCAGCCACTACCTTCTTGCCG	
Mouse	Ngb	F: CCACATTAGGAAGGTGATGCTAG	133
		R: CGAGAAGGAGCTGAGCCTC	
	Cygb	F: CATCCTGGTGAGGTTCTTTGTGA	160
		R: ATGCAGGTTCTCCACGACAGT	
	Mb	F: GGGGAGTGGCAGCTGGTGCTGA	199
		R: CTGTGAGCACGGTGCAACCATGC	
	Hba	F: GGTCGCCGATGCTCTGGCCAAT	125
		R: GGCTCAGGAGCTTGAAGTTGACGG	
Human	Ngb	F: CTGTTTGCCAGGCTGTTTGCC	154
		R: GGTCACTGCAGCATCAATCACG	
	Cygb	F: CCATCCTGGTGAGGTTCTTTGTG	122
		R: CGGCAGGCGTGCTTCCGCAG	
	Mb	F: GTCCTCATCAGGCTCTTTAAGGG	128
		R: GTGAGCACGGTGGCACCAT	
	Hba	F: GGCCCTGGAGAGGATGTTCCT	128
		R: CGGCGTTGGTCAGCGCGTCG	

## Results and Discussion

### Globin gene expression in nucleated vertebrate RBCs of fish and amphibians

**Figure 1:**
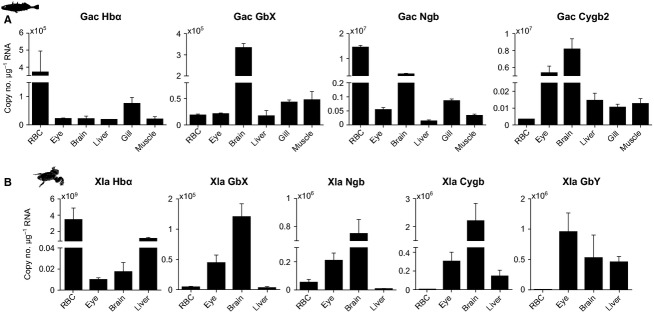
Absolute quantification (copies of transcripts per *μ*g RNA) of mRNA expression of globins in different tissues of (A) stickleback (Gac; *n *=* *3) and (B) clawed frog (Xla; *n *=* *3 or 4). Values are means ± SEM.

In three-spined stickleback (*Gasterosteus aculeatus*) we analyzed, if four (*Hbα*, *Cygb2*, *Ngb*, and *GbX*) of the five known teleost globin genes are transcribed in RBCs. Transcripts of all analyzed globins were detected in the examined tissues in various quantities (Fig.1A). Unexpectedly, the *Ngb* gene was transcribed in RBCs at even a higher level than *Hbα* and thus showed the highest copy numbers of all stickleback tissues. The exact function of Ngb remains yet unknown, but it is suggested to be involved in the oxygen tolerance of neuronal tissues (e.g., Burmester et al. 2000; Burmester and Hankeln 2009) due to the fact that Ngb mRNA and protein are upregulated after hypoxia/anoxia and reoxygenation in fish and turtles (Roesner et al. 2006; Nayak et al. 2009; Tiedke et al. 2014). The translational efficiency of the novel members of the globin superfamily has not yet been studied but it might be much lower than that of Hb. In contrast to Hb, which has a pivotal role in tissue oxygen delivery and thus is vital for the animal's survival, Ngb and other globins probably have only minor or indirect functions in oxygen transport in RBCs.

In contrast to other vertebrate species, teleost fish have two Cygb gene copies resulting from whole-genome duplication. *Cygb1* is transcribed in a broad range of tissues, whereas *Cygb2* mRNA is predominantly found in neuronal tissues (brain, eye) (Table2; Fuchs et al. 2005). In stickleback, only *Cygb2* mRNA could be found (Fig.1A), although the Cygb1 gene is represented in the genome (Ensemble gene accession number ENSGACG00000012736). With the exception of the *Ngb* mRNA expression in RBCs, the overall expressions in tissues and organs are broadly consistent with the mRNA expression patterns recently described from various fish species (see Table2 and references therein). In sticklebacks, myoglobin (Mb), the oxygen storage globin of the heart and skeletal muscles, shows pseudogenization and is thus not expressed (Macqueen et al. 2014). Amphibians possess, in addition to Hb, four other globins: Ngb, Cygb, GbX, and globin Y (GbY) (Fig.1B). Mb has been lost secondarily in Amphibia, including the African clawed frog *Xenopus laevis* (Xi et al. 2007). We found moderate transcript numbers from *Hbα*, *Ngb*, and *GbX* in RBCs of *X. laevis*, with *Hbα* being the dominant globin transcribed. For *Cygb* and *GbY*, only faint mRNA expressions in erythrocytes were observed. In agreement with their expression in a broad range of tissues (Table2) (Fuchs et al. 2006; Xi et al. 2007), we found comparably higher transcript copy numbers in the frogs' eye, brain, and liver.

**Table 2:** Literature survey of the tissue-specific transcription of globin genes in different vertebrate species

Species	Tissue-specific expression	Reference
Zebrafish (*Danio rerio*)	GbX: highest levels in brain and eye; low levels in muscle, liver, heart, and gill	Blank et al. (2011b)
Zebrafish (*Danio rerio*) & Pufferfish (*Tetraodon nigroviridis*)	Cygb 1: highest levels in brain, heart, eye, gut; Cygb 2: highest levels in brain, eye (250–300 fold higher than Cygb1), Cygb2 is higher expressed than Cygb1 with exception of gut and gills	Fuchs et al. (2005)
Goldfish (*Carassius auratus*)	GbX: weak signals in gills, muscle, heart, gut, kidney, spleen, liver; no expression in brain, eye	Roesner et al. (2005)
African clawed frog (*Xenopus laevis*)	GbX: highest in eye, weak in ovary, brain, heart, liver, kidney; no expression in skeletal muscle Ngb: predominantly in eye, brain; weaker in gut, ovary, kidney; not expressed in liver, heart, and skeletal muscle Cygb: in all tissues, highest in brain, kidney, eye GbY: in all tissues, strongest in ovary, kidney, eye; only faint expression in skeletal muscle	Fuchs et al. (2006)
African clawed frog (*Xenopus laevis*)	Cygb: highest levels in heart and skeletal muscle, weak in liver and spleen	Xi et al. (2007)
Western clawed frog (*Xenopus tropicalis*)	Ngb: in eye, gut, ovary, no expression heart, liver GbX: in eye, brain, gut, heart, no expression in liver, ovary, skeletal muscle	Fuchs et al. (2006)
Chicken (*Gallus gallus*)	Cygb: in all tissues (brain, eye, muscle, liver, spleen, heart) Ngb: only in brain, eye GbE: only in eye	Kakhniashvili et al. (2004)

### Globin E transcripts in bird RBCs

**Figure 2:**
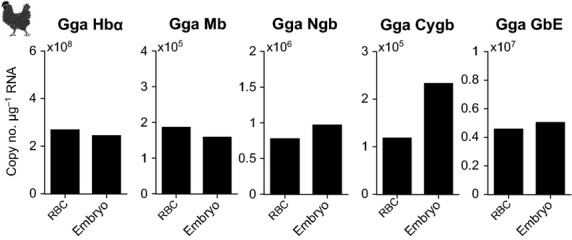
Absolute quantification (copies of transcripts per *μ*g RNA) of mRNA expression of globins in chicken (Gga) embryo blood and whole chicken embryo. Values are means of two individuals.

For the analysis of globin gene transcription in avian blood, 17-day-old (days post fertilization) chicken embryos were used. Tissue RNA was extracted from whole embryos and blood RNA from chicken embryo blood. At this stage of embryogenesis, a functional circulatory system and eyes are already developed. Transcripts of all five known avian globin genes (*Hbα*, *Mb*, *Cygb*, *Ngb*, and *GbE*) are represented in the blood (Fig.2). The levels were comparable to those in whole embryo preparations. A study on adult chicken showed that, with the exception of *Cygb*, all other globin genes are tissue specifically expressed (Kugelstadt et al. 2004). This is especially true for the eye-specific globin E gene (*GbE*) which is only transcribed in the eye of adult birds (Table2). Thus, the protein is suggested to be involved in the oxygen supply of this metabolically highly active tissue (Blank et al. 2011a). From the previously published results, it was expected that in RBCs *GbE* is either not transcribed at all or much less than in whole embryos. Our results are therefore surprising and further studies might reveal if *GbE* transcription switches during ontogenesis from a broader expression to an exclusive transcription in the eyes. However, based on the present results, *GbE* may be transcribed also in blood of mature birds.

During ontogenetic development many vertebrate species, including mammals, undergo one or even two maturational globin gene switches which are complex processes of gene activation and silencing and which allow expression of distinct globin genes at specific developmental stages (Ganis et al. 2012), reviewed in (Nikinmaa 1990). In chicken, the Hb globin switch from primitive to definitive blood occurs between days 3 and 6; and from day 7 on globin gene expression resembles that of adult animals (Alev et al. 2009). Nothing is known about the translational and transcriptional control of gene expression during development and maturation in other members of the globin superfamily so far. It is, however, conceivable that the expression of these globin genes in RBCs might also be time dependently associated with erythropoiesis and the age of the erythrocytes. In mammals, Hb gene expression is tightly regulated during erythropoiesis and terminal differentiation since this is an indispensable prerequisite for proper RBC development and function (Hardison 2012).

### Comparison of globin mRNA expression in two mammalian species

**Figure 3:**
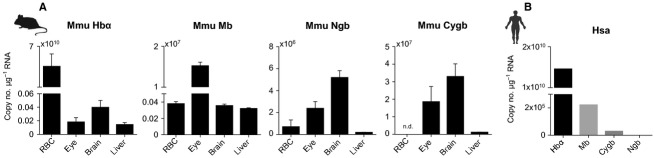
Absolute quantification (copies of transcripts per *μ*g RNA) of mRNA expression of globins in different tissues of (A) mouse (Mmu; *n *=* *3 or 4) and in red blood cells of (B) human (Hsa; *n *=* *1 or 2 independent samples of the same individual). Values are means ± SEM.

Mature anucleated RBCs of mammals are necessarily transcriptionally inactive. It has been postulated that at the final stage of erythroid differentiation, all mRNAs, including those of globins, are cleared from the cells before the mature reticulocytes are released into the circulation (Morales et al. 1997). In contradiction to that, microarray and proteomic studies of human peripheral red blood cells have revealed the presence of transcripts for 1019 genes (Kabanova et al. 2009) and up to 2289 proteins (Copeland et al. 1982; Kakhniashvili et al. 2004; Tyan et al. 2005), recently reviewed in (Goodman et al. 2013), but non-Hb-related proteins have not been found. Our comparative analysis of two mammalian species, human, and mouse also revealed globin transcripts in circulating erythrocytes of both species (Fig.3A and B).

Although mice have close genetic and physiological relationships to humans, slight differences in the mRNA expression of globin genes between species were found: *Hb* and *Mb* are transcribed in RBCs of both mammals. *Cygb* mRNA was only found in human RBCs, *Ngb* mRNA was present in mice but not in human RBCs, although both globin genes are transcribed in other tissues of both species. We compared our findings to a human erythrocyte transcriptome dataset provided by Kabanova et al. (2009) (available online in Gene Expression Omnibus database under accession no. GSE3674). These searches revealed that the dataset contained apart from different *Hbs*, also transcripts of *Mb* and *Ngb*; only *Cygb* was not present. This corresponds to recent results presented by D'Aprile et al. (2014) who found expression of *Mb* and *Ngb* in adult human hematopoietic stem/progenitor cells. Another online human gene database (GeneCards, www.genecards.org) displays mRNA expression of *Mb*, *Ngb*, and *Cygb* in blood plasma (Rebhan et al. 1998). At present, we have no explanation for the discrepancy between the datasets. Apparently, mRNA expression of globin genes shows either interindividual variability or other (unknown) factors are responsible for this difference.

In line with these recent studies, our results provide unequivocal evidence that even in anucleated RBCs transcripts of different globin genes are present indicating the long stability of the mRNA. Several reasons why *Mb*, *Cygb* and *Ngb* have not been found earlier in transcriptomic and proteomic studies are conceivable and might be related either to improper sample preparation procedures or to the fact that these globins have been simply overlooked in RBCs. Whole-blood sample preparation procedures require careful planning beforehand, since bias might be introduced, for example, by using (hemo-)globin purification kits, which not only remove mRNA and proteins of the highly abundant Hb but also of other hemoproteins.

## Conclusions

Our results demonstrate for the first time that mature circulating erythrocytes of all vertebrates not only contain *Hb* transcripts but also variable levels of other transcripts of the vertebrate globin family. Thus, RBCs are currently the only known tissue where the whole-globin repertoire of an animal is expressed. This adds new and unexpected aspects to the current knowledge on the expression specificity of globins in vertebrate tissues and organs. Although our study is limited to transcript levels, which do not necessarily reflect globin protein levels, several essential questions arise from the results. Thus, the study may be a starting point for further research, especially on globin protein expression and its functional significance in RBCs of nonmammalian and mammalian species. The functions of Hb and Mb are well understood and known since several years, but data about the factors controlling the expression of these ‘novel’ globins as well as on their physiological functions are fragmentary and often speculative. Compared to mammalian erythrocytes which are an intensively investigated model since many years, far less is known about nucleated RBCs of fish, amphibians, and birds. Further studies are thus required to unravel the factors determining globin mRNA stability, gene transcription, and translation.

## References

[b1] Alev C, Shinmyozu K, McIntyre BA, Sheng G (2009). Genomic organization of zebra finch alpha and beta globin genes and their expression in primitive and definitive blood in comparison with globins in chicken. Dev. Genes. Evol.

[b2] Bastos RN, Volloch Z, Aviv H (1977). Messenger RNA population analysis during erythroid differentiation: a kinetic approach. J. Mol. Biol.

[b3] Blank M, Burmester T (2012). Widespread occurrence of N-terminal acylation in animal globins and possible origin of respiratory globins from a membrane-bound ancestor. Mol. Biol. Evol.

[b4] Blank M, Kiger L, Thielebein A, Gerlach F, Hankeln T, Marden M (2011a). Oxygen supply from the bird's eye perspective: globin E is a respiratory protein in the chicken retina. J. Biol. Chem.

[b5] Blank M, Wollberg J, Gerlach F, Reimann K, Roesner A, Hankeln T (2011b). A membrane-bound vertebrate globin. PLoS One.

[b6] Burmester T, Hankeln T (2009). What is the function of neuroglobin?. J. Exp. Biol.

[b7] Burmester T, Hankeln T (2014). Function and evolution of vertebrate globins. Acta Physiol.

[b8] Burmester T, Weich B, Reinhardt S, Hankeln T (2000). A vertebrate globin expressed in the brain. Nature.

[b9] Copeland BR, Todd SA, Furlong CE (1982). High resolution two-dimensional gel electrophoresis of human erythrocyte membrane proteins. Am. J. Hum. Genet.

[b10] D'Aprile A, Scrima R, Quarato G, Tataranni T, Falzetti F, Di Ianni M (2014). Hematopoietic stem/progenitor cells express myoglobin and neuroglobin: adaptation to hypoxia or prevention from oxidative stress?. Stem Cells.

[b11] Dickerson AE, Geis I (1983). Hemoglobin: structure, function, evolution and pathology.

[b12] Fuchs C, Luckhardt A, Gerlach F, Burmester T, Hankeln T (2005). Duplicated cytoglobin genes in teleost fishes. Biochem. Biophys. Res. Commun.

[b13] Fuchs C, Burmester T, Hankeln T (2006). The amphibian globin gene repertoire as revealed by the *Xenopus* genome. Cytogenet Genome Res.

[b14] Ganis JJ, Hsia N, Trompouki E, de Jong JL, DiBiase A, Lambert JS (2012). Zebrafish globin switching occurs in two developmental stages and is controlled by the LCR. Dev. Biol.

[b15] Goodman SR, Daescu O, Kakhniashvili DG, Zivanic M (2013). The proteomics and interactomics of human erythrocytes. Exp. Biol. Med.

[b16] Hardison RC (1996). A brief history of hemoglobins: plant, animal, protist, and bacteria. Proc. Natl Acad. Sci. USA.

[b17] Hardison RC (2012). Evolution of hemoglobin and its genes. Cold Spring Harb. Perspect Med.

[b18] Hoogewijs D, Ebner B, Germani F, Hoffmann FG, Fabrizius A, Moens L (2012). Androglobin: a chimeric globin in metazoans that is preferentially expressed in Mammalian testes. Mol. Biol. Evol.

[b19] Kabanova S, Kleinbongard P, Volkmer J, Andree B, Kelm M, Jax TW (2009). Gene expression analysis of human red blood cells. Int. J. Med. Sci.

[b20] Kakhniashvili DG, Goodman LA, Bulla SR (2004). The human erythrocyte proteome: analysis by ion trap mass spectrometry. Mol. Cell Proteomics.

[b21] Kugelstadt D, Haberkamp M, Hankeln T, Burmester T (2004). Neuroglobin, cytoglobin, and a novel, eye-specific globin from chicken. Biochem. Biophys. Res. Commun.

[b22] Macqueen DJ, Garcia de la serrana D, Johnston IA (2014). Cardiac myoglobin deficit has evolved repeatedly in teleost fishes. Biol. Lett.

[b23] Morales J, Russell JE, Liebhaber SA (1997). Destabilization of human *α*-globin mRNA by translation anti-termination is controlled during erythroid differentiation and is paralleled by phased shortening of the poly(A) tail. J. Biol. Chem.

[b24] Nayak G, Prentice HM, Milton SL (2009). Role of neuroglobin in regulating reactive oxygen species in the brain of the anoxia-tolerant turtle *Trachemys scripta*. J. Neurochem.

[b25] Nikinmaa M, Bradshaw SD, Burggren W, Heller HC, Ishii S, Langer H, Neuweiler G, Randall DJ (1990). Vertebrate red blood cells. Zoophysiology.

[b26] Rebhan M, Chalifa-Caspi V, Prilusky J, Lancet D (1998). GeneCards: a novel functional genomics compendium with automated data mining and query reformulation support. Bioinformatics.

[b27] Roesner A, Fuchs C, Hankeln T, Burmester T (2005). A globin gene of ancient evolutionary origin in lower vertebrates: evidence for two distinct globin families in animals. Mol. Biol. Evol.

[b28] Roesner A, Hankeln T, Burmester T (2006). Hypoxia induces a complex response of globin expression in zebrafish (*Danio rerio*. J. Exp. Biol.

[b29] Snyder GK, Sheafor BA (1999). Red blood cells: centerpiece in the evolution of the vertebrate circulatory system. Am. Zool.

[b30] Storz JF, Opazo JC, Hoffmann FG (2013). Gene duplication, genome duplication, and the functional diversification of vertebrate globins. Mol. Phylogenet. Evol.

[b31] Tiedke J, Thiel R, Burmester T (2014). Molecular response of estuarine fish to hypoxia: a comparative study with ruffe and flounder from field and laboratory. PLoS One.

[b32] Tyan YC, Jong SB, Liao JD, Liao PC, Yang MH, Liu CY (2005). Proteomic profiling of erythrocyte proteins by proteolytic digestion chip and identification using two-dimensional electrospray ionization tandem mass spectrometry. J. Proteome Res.

[b33] Vinogradov SN, Moens L (2008). Diversity of globin function: enzymatic, transport, storage, and sensing. J. Biol. Chem.

[b34] Xi Y, Obara M, Ishida Y, Ikeda S, Yoshizato K (2007). Gene expression and tissue distribution of cytoglobin and myoglobin in the Amphibia and Reptilia: possible compensation of myoglobin with cytoglobin in skeletal muscle cells of anurans that lack the myoglobin gene. Gene.

